# Circulating microRNA Profiling Identifies a Subset of Metastatic Prostate Cancer Patients with Evidence of Cancer-Associated Hypoxia

**DOI:** 10.1371/journal.pone.0069239

**Published:** 2013-07-30

**Authors:** Heather H. Cheng, Patrick S. Mitchell, Evan M. Kroh, Alexander E. Dowell, Lisly Chéry, Javed Siddiqui, Peter S. Nelson, Robert L. Vessella, Beatrice S. Knudsen, Arul M. Chinnaiyan, Kenneth J. Pienta, Colm Morrissey, Muneesh Tewari

**Affiliations:** 1 Clinical Research Division, Fred Hutchinson Cancer Research Center, Seattle, Washington, United States of America; 2 Department of Medicine, University of Washington, Seattle, Washington, United States of America; 3 Human Biology Division, Fred Hutchinson Cancer Research Center, Seattle, Washington, United States of America; 4 Department of Urology, University of Washington, Seattle, Washington, United States of America; 5 Michigan Center for Translational Pathology, University of Michigan Medical School, Ann Arbor, Michigan, United States of America; 6 Department of Pathology, University of Michigan Medical School, Ann Arbor, Michigan, United States of America; 7 Department of Veterans Affairs Medical Center, Seattle, Washington, United States of America; 8 Public Health Sciences Division, Fred Hutchinson Cancer Research Center, Seattle, Washington, United States of America; 9 Department of Urology, University of Michigan Medical School, Ann Arbor, Michigan, United States of America; 10 Bioinformatics Program, University of Michigan Medical School, Ann Arbor, Michigan, United States of America; 11 Departments of Urology, Oncology and Pharmacology and Molecular Sciences, Johns Hopkins Hospital, Baltimore, Maryland, United States of America; UCSF/VA Medical Center, United States of America

## Abstract

MicroRNAs (miRNAs) are small (∼22 nucleotide) non-coding RNAs that regulate a myriad of biological processes and are frequently dysregulated in cancer. Cancer-associated microRNAs have been detected in serum and plasma and hold promise as minimally invasive cancer biomarkers, potentially for assessing disease characteristics in patients with metastatic disease that is difficult to biopsy. Here we used miRNA profiling to identify cancer-associated miRNAs that are differentially expressed in sera from patients with metastatic castration resistant prostate cancer (mCRPC) as compared to healthy controls. Of 365 miRNAs profiled, we identified five serum miRNAs (miR-141, miR-200a, miR-200c, miR-210 and miR-375) that were elevated in cases compared to controls across two independent cohorts. One of these, miR-210, is a known transcriptional target of the hypoxia-responsive HIF-1α signaling pathway. Exposure of cultured prostate cancer cells to hypoxia led to induction of miR-210 and its release into the extracellular environment. Moreover, we found that serum miR-210 levels varied widely amongst mCRPC patients undergoing therapy, and correlated with treatment response as assessed by change in PSA. Our results suggest that (i) cancer-associated hypoxia is a frequent, previously under-appreciated characteristic of mCRPC, and (ii) serum miR-210 may be further developed as a predictive biomarker in patients with this distinct disease biology.

## Introduction

The natural history of patients with metastatic castration resistant prostate cancer (mCRPC) varies widely, suggesting a heterogeneous disease biology in this patient population. However, little is known about biological heterogeneity in mCRPC and approaches for clinical stratification of patients are limited, in large part because metastatic tissue is not routinely available for study. Minimally invasive (e.g., blood-based) approaches that can help stratify patients on the basis of distinct tumor biology could help inform choice of therapy, which is especially relevant with the recent introduction of multiple new effective treatments for mCRPC.

Circulating microRNAs (miRNAs) are an emerging class of blood-based biomarkers with the potential to provide information about distinct tumor biology in individual patients [1]. miRNAs are small (∼22 nucleotide), non-coding RNA molecules that post-transcriptionally regulate gene expression by translational repression or degradation of targeted transcripts [2]. Specific miRNAs have been found to regulate a variety of critical processes in tumor physiology, including angiogenesis [3], epithelial-to-mesenchymal transition [4], metastasis [5] and the tumor response to hypoxia [6]. miRNAs have been demonstrated to be effective tissue-based cancer biomarkers–in diagnosing cancers of unknown tissue origin and in predicting clinical outcomes[7]–[9]. We and others have demonstrated that circulating, cell-free, tumor-derived miRNAs are highly stable and detectable in the serum of cancer patients [1]. In earlier work, we used a candidate miRNA approach to identify significant elevation of miR-141 in serum from patients with metastatic castration-resistant prostate cancer (mCRPC) [1].

In order to more comprehensively identify prostate cancer-associated circulating miRNAs, in the current study we profiled serum miRNAs from patients with mCRPC. We identified five serum miRNAs that were significantly elevated in cases compared to healthy controls, including the hypoxia-associated miR-210. In order to determine whether prostate cancer cells can release miR-210 in response to hypoxia, we exposed prostate cancer cell lines to hypoxic conditions and found that miR-210 was induced and released into the extracellular environment. In analysis of clinical specimens and outcomes, we found evidence that a subset of mCRPC patients have increased miR-210 levels and that this correlates with treatment response, suggesting that increased hypoxia is a feature of mCRPC that may define a subset of patients with a distinct disease biology.

## Materials and Methods

### Cell Culture

LNCaP (ATCC® CRL-1740™) and VCaP [10] human prostate cancer cell lines were cultured in RPMI 1640 and DMEM, respectively, each supplemented with 10% FBS (or under serum-free conditions, as noted), at 37°C in a 5% CO_2_ incubator. Hypoxic conditions (1% O_2_) were established in a Thermo Scientific 3595 Incubator (ThermoFisher), with cells maintained under normoxic conditions (20% O_2_) in parallel.

### RNA Isolation from Cultured Cells and Conditioned Media

Conditioned media was removed from cells cultured for 24, 48 or 72 hours under normoxic or hypoxic conditions. Cells were washed with 5 ml PBS and lysed on ice directly in the culture dish with 600 µl Lysis/Binding buffer from the *mir*Vana miRNA isolation kit (Ambion). Lysates were harvested manually with a sterile cell scraper and transferred to an RNase−/DNase-free 2 ml microcentrifuge tube. RNA was extracted from cell lysates following the manufacturer’s recommended protocol for total RNA isolation. Cellular debris was removed from a 500 µl aliquot of conditioned media (10 ml total volume) by filtration through a 0.2 µm NanoSep filtration unit (Millipore) at 14,000×*g*, 5 min, at room temperature. 400 µl filtered sample was combined with 400 µl 2X Denaturing Solution (Ambion) and vortexed. *C. elegans* spiked-in oligonucleotides were introduced (as a mixture of 25 fmol of each oligonucleotide in 5 µl total volume per liquid sample) after denaturation and used for normalization of variability in RNA isolation across samples as previously described [1]. RNA was extracted from conditioned media lysates using the *mir*Vana PARIS kit (Ambion) following the manufacturer’s recommended protocol for total RNA isolation.

### Ethics Statement

All clinical samples were obtained from subjects who provided written informed consent. Studies were performed in accordance with the declaration of Helsinki guidelines and with ethics approval from the Institutional Review Boards at the University of Washington, Fred Hutchinson Cancer Research Center and University of Michigan.

### Blood Processing and Isolation of Serum from Clinical Samples

All clinical samples obtained at the University of Washington and the University of Michigan were collected and processed as previously described [1], [11].

### RNA Isolation from Clinical Serum Samples

Total RNA was isolated from 400 µl serum collected at the University of Washington using the mirVana PARIS kit (Ambion) as previously described [1]. Equal volumes of each sample type were pooled to create mCRPC and healthy donor serum pools (n = 25 for each pool) for TLDA profiling. Total RNA was isolated from serum samples collected at the University of Michigan using the miRNeasy RNA isolation kit (Qiagen) as follows: 400 µl serum was divided into four, 100 µl aliquots. Each aliquot was denatured using 10X volume (1 ml) Qiazol, which was vortexed and incubated at room temperature for 10 min. *C. elegans* spiked-in oligonucleotides were introduced (as a mixture of 25 fmol of each oligonucleotide in 5 µl total volume per liquid sample) after denaturation, which were used for normalization of variability in RNA isolation across samples as previously described [1]. RNA was extracted using 0.2X volume chloroform (220 µl), and total RNA was isolated following the manufacturer’s protocol. For a given sample, RNA isolated from each 100 µl aliquot was pooled and concentrated to 100 µl volume over Microcon YM-3 filter units (Millipore) at 14,000×*g*, 1.5 hour, 4°C, which were loaded inverted into pre-weighed 1.5 ml microcentrifuge tubes and eluted at 1000×*g*, 3 min, 4°C. Tubes plus eluate was weighed on an analytical scale and brought to 100 µl with Elution Buffer. RNA was stored at −80°C.

### Collection and Processing of Clinical Tissue Sections

#### Laser-capture micro-dissection (LCM) of frozen-tissue sections

Sections of flash-frozen prostate and lymph node obtained from radical prostatectomy and rapid autopsy, respectively, were assessed by a pathologist to define regions of tumor epithelial cells. For laser capture microdissection 5 µm sections of frozen tissue were made on a Leica™CM3050S cryostat at −20°C (Leica, Wetzlar, Germany), placed onto PEN Membrane Frame Slides (MDS Inc., Ontario, Canada) then stained and fixed according to the HistoGene™ LCM Frozen Section Staining Kit protocol (MDS Inc.). For each sample approximately 2000 cells were laser captured at 200x magnification (Veritas, Arcturus MDS Inc., Ontario, Canada). The captured sample was placed in 300 µl lysis buffer from the RNAqueous® RNA Isolation Kit (Ambion, Austin, Texas), incubated for 30 minutes at 42°C and stored at −80°C until RNA isolation was performed. miRNA was then isolated using the RNAqueous® RNA Isolation Kit (Ambion).

#### RNA isolation from LCM tissue samples

Total RNA was isolated from LCM tissue samples using the RNAqueous-Micro RNA isolation kit (Ambion) as follows: Frozen lysates were thawed on ice, vortexed and centrifuged at 16,100×g, 30 sec, room temperature. *C. elegans* spiked-in oligonucleotides were introduced (as a mixture of 25 fmol of each oligonucleotide in 5 µl total volume per liquid sample) and used for normalization of variability in RNA isolation across samples as previously described [1], followed by addition of 3 µl LCM Additive. RNA was precipitated from the lysate mixture with 1.25 volumes 100% molecular-grade EtOH, and was subsequently bound to the Micro Filter Cartridge assembly (prewet with 30 µl Lysis Solution for 5 min) by centrifugation at 10,000×g, 1 min. The filter was washed (180 µl Wash Solution 1, 10,000×*g*, 1 min; 2×180 µl Wash Solution 2/3, 16,100×*g*, 30 sec; air only, 16,100×*g*, 30 sec). RNA was eluted from column twice with 10 µl 95°C Elution Buffer into pre-weighed tubes. Eluates were weighed on an analytical scale and brought to 20 µl with Elution Buffer. RNA was stored at −80°C.

### MicroRNA Profiling using TaqMan Low-Density Array miRNA qRT-PCR and Biomarker Candidate Selection

RNA from pooled serum of mCRPC patients or healthy controls (comprised of equal RNA volume from each of 25 samples per pool) was reverse-transcribed in duplicate reactions from 2 µl of pooled RNA using the TaqMan miRNA Reverse Transcription Kit and the TaqMan miRNA Multiplex RT Assays (Human Pool Set). A pooled sample approach was chosen for cost-efficient discovery of miRNA biomarkers. miRNA expression was profiled from each RT reaction replicate using the TaqMan Low-Density Array (TLDA, v1.0) as previously described [1]. Multiplex reverse-transcription TLDA qRT-PCR was carried out on an Applied BioSystems 7900HT thermocycler using the manufacturer’s recommended cycling conditions. Data were analyzed with SDS Relative Quantification Software version 2.2.2 (Applied BioSystems), with an automatically assigned minimum threshold, which was above the baseline of all assays showing measurable amplification above background. Values that were below the minimum threshold were arbitrarily assigned a cycle threshold (CT) value of 40. *P*-values were assigned by Student’s t-test evaluating replicate profiling data from each pool to determine significant differences in miRNA expression between mCRPC pool and healthy control pool RNA samples. Fold-change (FC) values were derived by computing 2^∧^ (AveCT_mCRPC pool_-AveCT_healthy control pool_). MicroRNA candidate biomarkers were selected by criterion of FC >5 and *P*<0.05.

### Measurement of miRNA and mRNA Levels by Individual TaqMan Quantitative Reverse-Transcription PCR (qRT-PCR)

miRNA-derived from serum samples was reverse-transcribed using the TaqMan miRNA Reverse Transcription kit (Applied BioSystems) and quantified by TaqMan miRNA qRT-PCR using miRNA-specific primer/probe sets (Applied BioSystems) as previously described [1]. A complete description of TaqMan assays used in this study is provided in **Table S4**.

miRNAs derived from serum samples obtained from the University of Michigan were quantified by TaqMan miRNA qRT-PCR using synthetic miRNA standard curves for absolute quantification identically to that described for the University of Washington sample set [1] with the exception that the pre-amplification step was excluded from all miRNA quantification other than miR-210 (this was based on empiric evidence that pre-amplification does not increase the absolute copy number of miRNA detectable for other TaqMan miRNA assays used here, data not shown).

Gene-expression was quantified by TaqMan qRT-PCR as follows: input RNA was reverse transcribed using the TaqMan Gene-Expression Reverse Transcription Kit (Applied BioSystems) in a small-scale RT reaction [comprised of 0.5 µl of 10X Reverse-Transcription Buffer, 0.5 µl 10X Random Primers, 0.2 µl 100 mM dNTPs with dTTP, 0.25 µl Multiscribe Reverse-Transcriptase and 3.55 µl input RNA; components other than the input RNA were prepared as a larger volume master mix], using a Tetrad2 Peltier Thermal Cycler (BioRad) at 50°C for 2 min, followed by 30 cycles of 95°C for 10 min, 95°C for 15 sec and 60°C for 60 sec. Resultant cDNA was combined with 3.75 µl pre-amplification assay reagents [comprised of 2.5 µl TaqMan PreAmp Master Mix and 1.25 µl TaqMan Gene-Expression Assay (GUSB or KRT18) diluted 1∶100 in TE; components other than the input cDNA were prepared as a larger volume master mix to generate a 5.0 µl total volume PCR reaction], using a Tetrad2 Peltier Thermal Cycler (BioRad) at 95°C for 10 min followed by 14 cycles of 95°C for 15 sec and 60°C for 4 min. GUSB or KRT18 pre-amplification reaction products were combined with 2.75 µl of PCR assay reagents [comprised of 2.5 µl TaqMan 2X Universal PCR Master Mix, No AmpErase UNG and 0.25 µl 20X TaqMan Gene-Expression Assay) to generate a 5.0 µl total volume PCR reaction for GUSB or KRT18, respectively. Real-time PCR was carried out on an Applied BioSystems 7900HT thermocycler at 95°C for 10 min, followed by 40 cycles of 95°C for 15 sec and 60°C for 1 min. Data were analyzed with SDS Relative Quantification Software version 2.2.2 (Applied BioSystems), with the automatic CT setting for assigning baseline and threshold for CT determination (See **Table S3** for results of messenger RNA measurements).

## Results and Discussion

**Figure 1:**
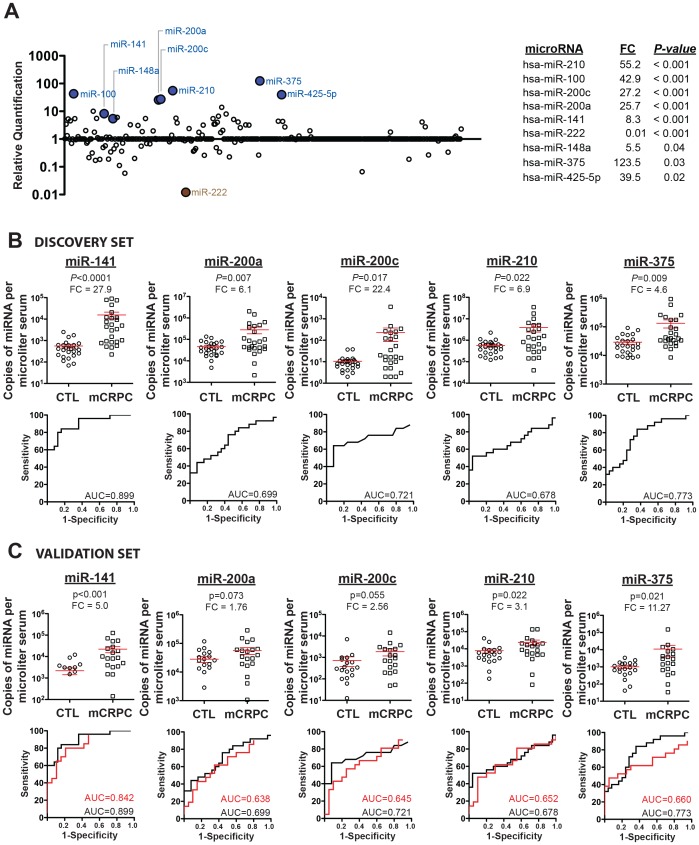
Serum miRNA profiling and validation. (*A)* Measurement of circulating miRNAs in sera pooled from patients with advanced prostate cancer as compared to healthy donors (comprising a Discovery Set) by TLDA profiling. Blue- and brown-filled circles represent serum miRNAs increased or decreased (with unadjusted p-value <0.05), respectively, in mCRPC patients compared to healthy controls. *Inset:* Nine miRNAs demonstrated >5-fold change (unadjusted *P*<0.05, Student’s t-test). FC, fold-change. (*B)* Confirmation of mCRPC-associated serum miRNAs in individual samples from the Discovery Set from the University of Washington samples. *Upper*: miRNA biomarker candidates were measured in individual samples by TaqMan miRNA qRT-PCR (*P* value assigned by Wilcoxon signed-rank test), where miRNA abundance is given in terms of miRNA copies/µl serum. Red bars, mean +/− SEM of miRNA copies/µl serum for each group. *Lower*: Receiver operating characteristic (ROC) curves plot sensitivity vs. (1 - specificity) to assess the ability of each miRNA biomarker to distinguish cases from controls. *(C)* Validation of mCRPC-associated serum miRNAs in an independent Validation Set. *Upper*: Serum concentration (copies/µl) of miR-141, miR-375, miR-200c, miR-200a and miR-210 was measured by TaqMan miRNA qRT-PCR. Dot-plot associated *P* values were assigned by Wilcoxon signed-rank test. Dot plots and ROC curves were generated as described for **Fig. 1**. *Lower*: *Red*, results from the validation sample set obtained from the University of Michigan. *Black*, results from the primary sample set obtained from the University of Washington reproduced from **Fig. 1** ***B*** *, lower*. AUC, area under the curve; mCRPC, prostate cancer patient sera; FC, fold-change; CTL, control sera (from age-matched male individuals with normal PSA and negative digital rectal exam).

In order to efficiently discover serum miRNAs that are differentially abundant between cancer cases vs. controls, we used real-time PCR-based miRNA TaqMan Low-Density Arrays (TLDA) to screen for differential abundance of 365 miRNAs in serum RNA pooled from mCRPC patients (*n = *25) versus age-matched controls (*n = *25; all controls had normal PSA and normal digital rectal exam findings). After normalization of data using spike-in control miRNAs and comparison of pooled serum RNA from mCRPC cases to that of controls (**Fig. 1** ***A*** and *inset*), we identified nine miRNAs demonstrating a greater than 5-fold change in abundance (unadjusted *P*<0.05, Student’s t-test). We next individually measured these nine miRNAs from the serum RNA of individual mCRPC cases and controls using miRNA-specific Taqman qRT-PCR assays. Serum levels of five miRNAs were confirmed to be significantly elevated in mCRPC cases compared with controls (miR-141: *P*<0.0001, miR-200a: *P = *0.007, miR-200c: *P = *0.017, miR-375: *P = *0.009, and miR-210: *P = *0.022, Wilcoxon signed-rank analysis). The average fold-difference between cases and controls ranged from 4.6 (miR-375) to 27.9 (miR-141) (**Fig. 1** ***B***, *upper* and **Table S1**). In addition, receiver-operating characteristic (ROC) plots demonstrate the capacity of these miRNAs to discriminate between the two groups (miR-141 Area Under the Curve (AUC) = 0.899; miR-200a AUC = 0.699; miR-375 AUC = 0.773; miR-200c AUC = 0.721 and miR-210 AUC = 0.678) (**Fig. 1** ***B***, *lower*). Importantly, we verified that control miRNAs were not differentially expressed between the two populations (**Fig. S1)**.

To validate these findings in an independent specimen set collected at a different institution, we measured miR-141, miR-200a, miR-200c, miR-375 and miR-210 from the sera of an additional 21 mCRPC patients and 20 age-matched healthy controls collected at the University of Michigan. All five miRNAs were elevated in sera from mCRPC cases relative to controls in this independent validation set. MiR-141, miR-375 and miR-210 were significant at a *P*-value threshold of <0.01 in the second cohort (*P = *0.001, *P = *0.021, *P = *0.022, respectively) and miR-200a and miR-200c tended toward significance (*P = *0.073, *P = *0.055, respectively) (**Fig. 1** ***C***, *upper*). ROC curves were generally concordant between the specimen sets from the two institutions (**Fig. 1** ***C***, *lower*). Analysis of serum miRNA markers in various combinations demonstrated that adding additional miRNAs to serum miR-141 (which had the best performance alone) did not improve the ability to distinguish between cases and controls (data not shown). Consistent with this observation, we found that among cancer cases in which expression of miR-141, miR-200a, miR-200c, and miR-375 was higher than all healthy controls, these miRNAs were also significantly correlated with each other and with serum PSA (**Table S2**). In contrast, miR-210 did not show significant correlation with any of these four miRNAs (**Table S3**) nor with serum PSA, suggesting that it provides distinct information about disease biology.

To determine whether the five serum miRNA markers of mCRPC are expressed in prostate cancer tissue and could therefore be plausibly cancer cell-derived, we measured their expression in epithelial cells that had been laser capture micro-dissected from primary prostate cancer tissues (*n = *8) and lymph node metastases (*n = *8). We detected miR-141, miR-200a, miR-200c, miR-375 and miR-210 in all tissue types evaluated (**Table S3**), suggesting that these five miRNAs, when found in the circulation, may originate from prostate cancer, although other additional sources cannot be excluded.

Three of the serum prostate cancer-associated miRNAs identified (miR-141, miR-200a and miR-200c) are epithelial-specific, highly related in sequence and have known roles in maintaining the epithelial state by suppression of the epithelial-to-mesenchymal transition [4]. We hypothesize that elevated circulating levels of miR-141, miR-200a and miR-200c reflect the epithelial origin of prostate cancer cells.

The presence of elevated circulating miR-141 and miR-375 in mCRPC patients has also been observed in recent mCRPC circulating miRNA biomarker studies [12]–[16]. Interestingly, elevated miR-210 was not reported in these other studies, despite the fact that we observed this in independent specimen sets from two different institutions. This could be due to different comparison groups used (e.g., localized prostate cancer rather than healthy controls as the comparator to mCRPC), the use of plasma rather than serum, differences in the data analytic approach used to identify differentially expressed miRNAs, as well as potential differences in the clinical characteristics of the mCRPC patients across different studies.

The elevated levels of miR-210 in serum from patients with mCRPC was particularly interesting because this miRNA is well-known to be transcriptionally activated by the hypoxia-inducible factor 1 alpha (HIF-1α) [17], [18] and may contribute to adaptation to hypoxia in tumors [19], [20]. This raises the possibility that miR-210 is produced and released by hypoxic cells in the prostate cancer (and/or by the tumor microenvironment), a potential explanation for elevated levels of miR-210 we observed in the serum of a subset of patients with mCRPC.

**Figure 2:**
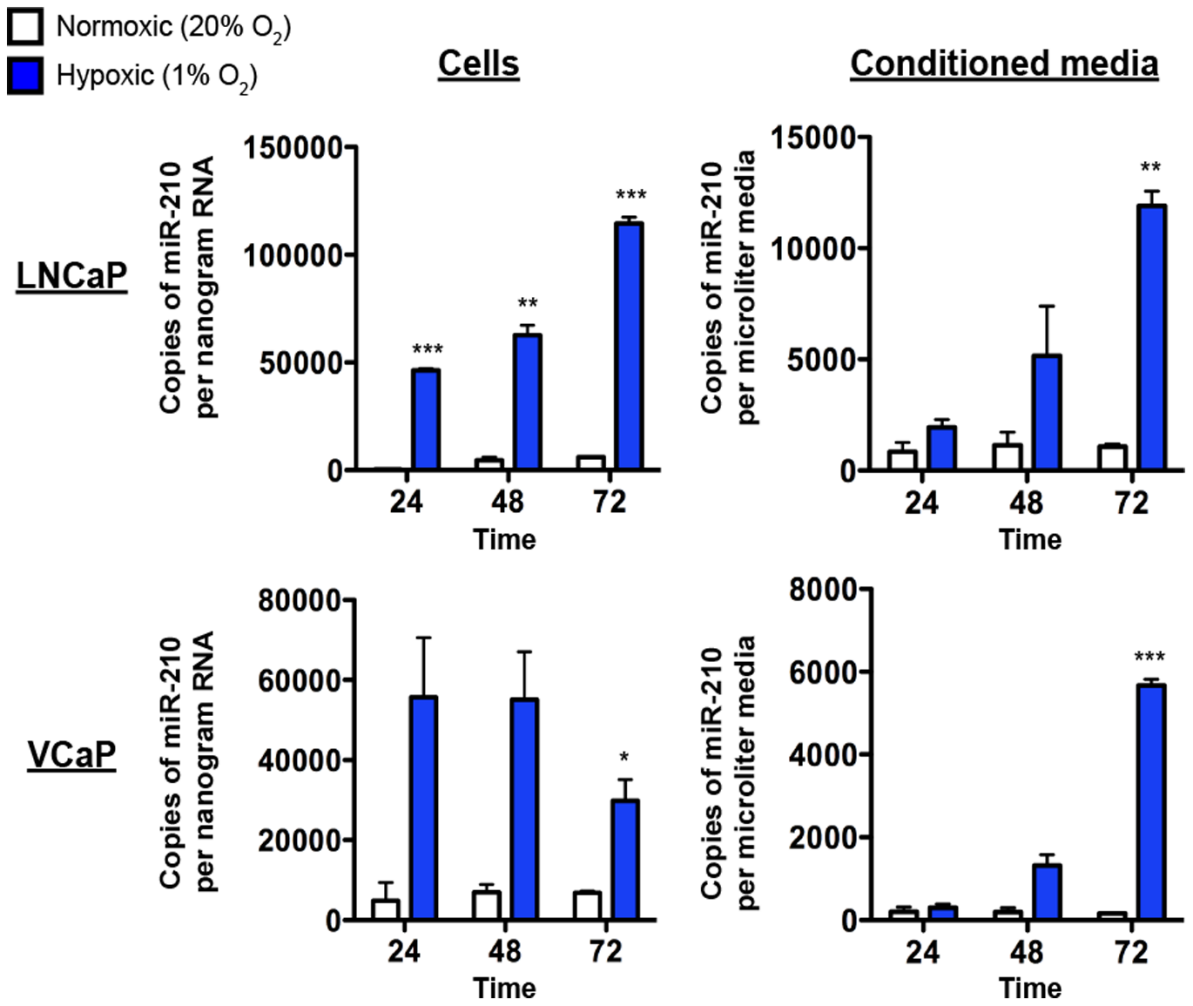
Exposure of prostate cancer cell lines to hypoxia induces production and release of miR-210 into the extracellular environment. *Left column,* miR-210 copies/ng RNA in LNCaP and VCaP human prostate cancer cell lines cultured in normoxic (20% O_2_) (white bars) or hypoxic (1% O_2_) (blue bars) conditions for 24, 48 or 72 hours. *Right column,* miR-210 copies/µl in filtered conditioned media corresponding to cellular samples. *, *P* value <0.05; **, *P* value <0.01; ***, *P* value <0.001 (Student’s t-test).

To test whether hypoxia can stimulate production and release of miR-210 in prostate cancer cells, we characterized miR-210 abundance in LNCaP and VCaP prostate cancer cell lines (as well as in filtered conditioned media) under normoxic (20% O_2_) and hypoxic (1% O_2_) conditions over a 72-hour time course (**Fig. 2**). miR-210 levels were increased by hypoxia compared to normoxia with an initial induction in LNCaP cells followed by a subsequent increased level in the conditioned media (**Fig. 2**). In VCaP cells, we did not observe the same increase in miR-210 copies/ng of RNA and the levels dropped at 72 hours. We speculate that this could be due to cell death or, alternatively, that the regulation of miR-210 in response to hypoxia in VCaP cells may be primarily occurring at the level of release. However, we did observe a stepwise, time-dependent increase in the level of extracellular miR-210 in the conditioned media of VCaP cells (**Fig. 2**). Taken together, the results indicate that elevated levels of miR-210 detected in serum could reflect tumor hypoxia.

Tumor hypoxia is a well-characterized process that contributes to cancer progression and metastasis in many human cancers [21]. Evaluation of tumor hypoxia in mCRPC has been limited to date due to infrequent sampling of metastases for routine clinical care. In an immunohistochemistry study of HIF-1α expression that incorporated a small set of prostate cancer metastases, HIF-1α expression was observed to vary widely in metastatic lesions [22]. Here, we show that a subset of patients with metastatic prostate cancer have increased levels of serum miR-210, providing evidence for previously under-appreciated hypoxia in mCRPC. Although non-tumor tissue sources of miR-210 cannot be ruled out, the fact that systemic hypoxemia is not a typical feature of mCRPC is consistent with a model in which tumor tissue hypoxia is the origin of the excess serum miR-210. Notably, elevated circulating miR-210 has also been observed in patients with pancreatic adenocarcinoma [23], a disease in which tumor hypoxia is well-recognized and is due to high interstitial pressure due to the host desmoplastic response.

**Figure 3:**
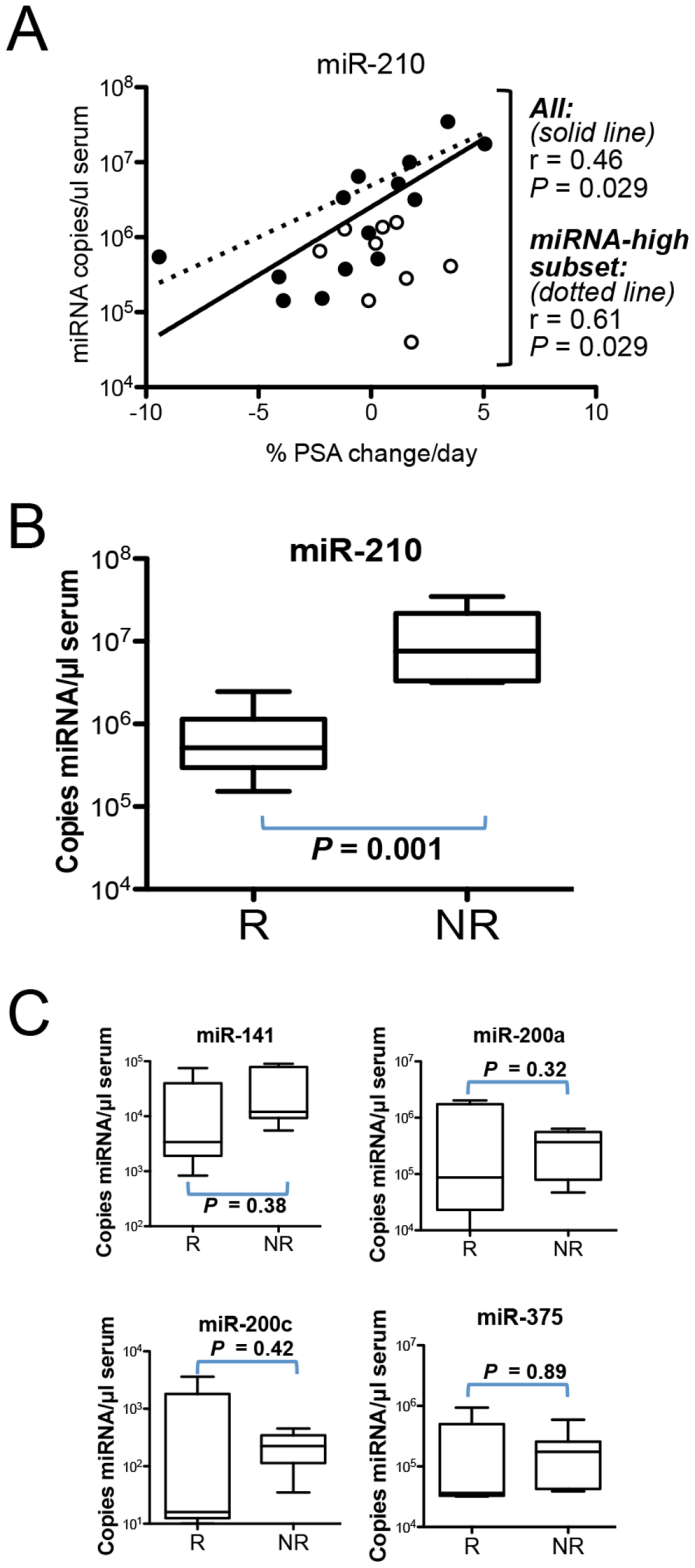
Relationship between serum miR-210 levels and PSA response in patients with metastatic castration resistant prostate cancer. *Upper:* miR-210 copies/µl serum versus %PSA change/day. %PSA change/day represents a measure of response to treatment and was calculated using available clinical PSA values measured most recently prior to, and at the time of serum miR-210 draw. Mean time elapsed between the two blood draws was 30 days. Closed circles represent the subset of patients defined as "miRNA-high" based on higher abundance of mCRPC-associated serum miRNAs compared to all control individuals (as described in Results and Discussion). Open circles represent the patients with mCRPC who did not meet the definition for “miRNA-high”. Solid line represents trend line of the miRNA-high patient subset, dotted line represents trend line of all patients. *Middle:* miR-210 copies/µl serum in patients with either a PSA Response (R) or No PSA Response (NR). PSA Response is defined as a decreasing or stable PSA (any change less than a 25% increase), and No PSA Response is defined as a PSA increase of 25% or more, similar to the Prostate Cancer Working Group criteria. *Lower:* Copies/µl serum of miR-141, miR-200a, miR-200c and miR-375 in patients, R and NR.

A well-documented phenomenon associated with tumor hypoxia is the association with resistance to treatment with radiotherapy, chemotherapy and other therapies [21]. To determine whether observed serum miR-210 levels were associated with treatment resistance, we retrospectively assessed whether patients were responding or resistant to ongoing therapy by calculating %PSA change/day using available clinical PSA values measured most recently prior to and at the time of serum miR-210 draw. Therapies varied among patients in this retrospective population, but typically involved androgen deprivation therapy using a GnRH agonist in combination with a chemotherapeutic agent (e.g., docetaxel, mitoxantrone). We found that serum miR-210 levels were significantly correlated with %PSA change/day during treatment (**Fig. 3** ***A*** **,** Pearson r = 0.46, *P = *0.029). To reduce potential noise from patients who are less informative due to low levels of cancer-associated serum miRNAs, we also analyzed a subset of patients with high levels of mCRPC-associated serum miRNAs (i.e., “miRNA-high subset”, defined as patients whose serum miR-141, miR-200a, miR-200c and/or miR-375 levels were greater than the highest value observed in any of the 25 healthy controls). In this group, the correlation between serum miR-210 and %PSA change/day was even stronger (**Fig. 3** ***A*** **,** Pearson r = 0.61, *P* = 0.029). Furthermore, serum levels of miR-210 were strikingly lower in patients whose disease was responding to treatment (PSA stable or decreasing), as compared to those whose disease was resistant to treatment (PSA increasing by ≥25%) (**Fig. 3** ***B, ****P* *= *0.001). Importantly, we did not observe this association with the other four serum miRNAs identified in our study (**Fig. 3** ***C***). Our data suggests a model in which increased hypoxia response signaling is present in a subset of mCRPC patients, leading to increased serum miR-210 and therapy resistance.

To our knowledge, this is the first report of circulating miR-210 in association with mCRPC. Our results raise the possibility that serum miR-210 levels could be used to identify a biologically distinct, subset of mCRPC patients with tumor-associated hypoxia for whom the development of alternative therapeutic approaches could be considered. For example, plasma miR-210 levels have been reported to be elevated in pancreatic cancer patients and as an indicator of hypoxia [23], [24], as well as correlated with response to trastuzumab in breast cancer patients [25]. In addition, mTOR inhibitors are being studied in prostate cancer, and pre-clinical studies have shown that mTOR inhibition can lead to AKT activation and HIF-1α transcriptional activation [26]. In this context, we speculate that elevated serum miR-210 could have potential utility as a predictive or response biomarker for this class of therapeutics. In addition, it will be important in future studies to determine whether miR-210 is not only an indicator of hypoxia and aggressive biology, but also an active mediator of an aggressive disease phenotype in mCRPC patients.

Given that the number of new agents effective against mCRPC is increasing, minimally invasive approaches such as serum miR-210 analysis may lead to clinical decision aids that can differentiate and help guide treatment decisions by differentiating between biologically distinct disease subtypes. This could be particularly important in settings where PSA is less informative, such as in neuroendocrine differentiated subtypes, or when cancers progress to an androgen pathway independent state.

## Supporting Information

Figure S1 Negative control miRNAs are not significantly different in abundance between mCRPC patients and healthy controls. *(A)* miRNAs were measured in individual samples by TaqMan miRNA qRT-PCR (*P* value assigned by Wilcoxon signed-rank test), where miRNA abundance is given in terms of miRNA copies/µl serum. Red bars, mean +/− SEM of miRNA copies/µl serum for each group. *(B)* Receiver operating characteristic (ROC) curves plot sensitivity vs. (1 - specificity) to assess the ability of each serum miRNA to distinguish mCRPC and control sera.
(TIF)

Table S1 Validation of candidate microRNA biomarkers in serum from mCRPC patients and healthy controls by single-plex microRNA TaqMan qRT-PCR. (TIF)

Table S2 Correlation analysis of mCRPC-associated serum miRNAs with each other and with serum PSA. (TIF)

Table S3 Results of measurement of mCRPC-associated serum microRNA markers and endogenous controls in microdissected laser-captured primary prostate cancer (“Cancer”) and lymph node metastases (“LN Met”) tissue. (TIF)

Table S4 Single-plex TaqMan assays used in this study. (PDF)
