# Supplementary figures and images for: Circulating microRNA Profiling Identifies a Subset of Metastatic Prostate Cancer Patients with Evidence of Cancer-Associated Hypoxia
Source: PLoS One. 2013 Jul 30;8(7):e69239. doi: 10.1371/journal.pone.0069239 (PMC3728349; doi:10.1371/journal.pone.0069239)

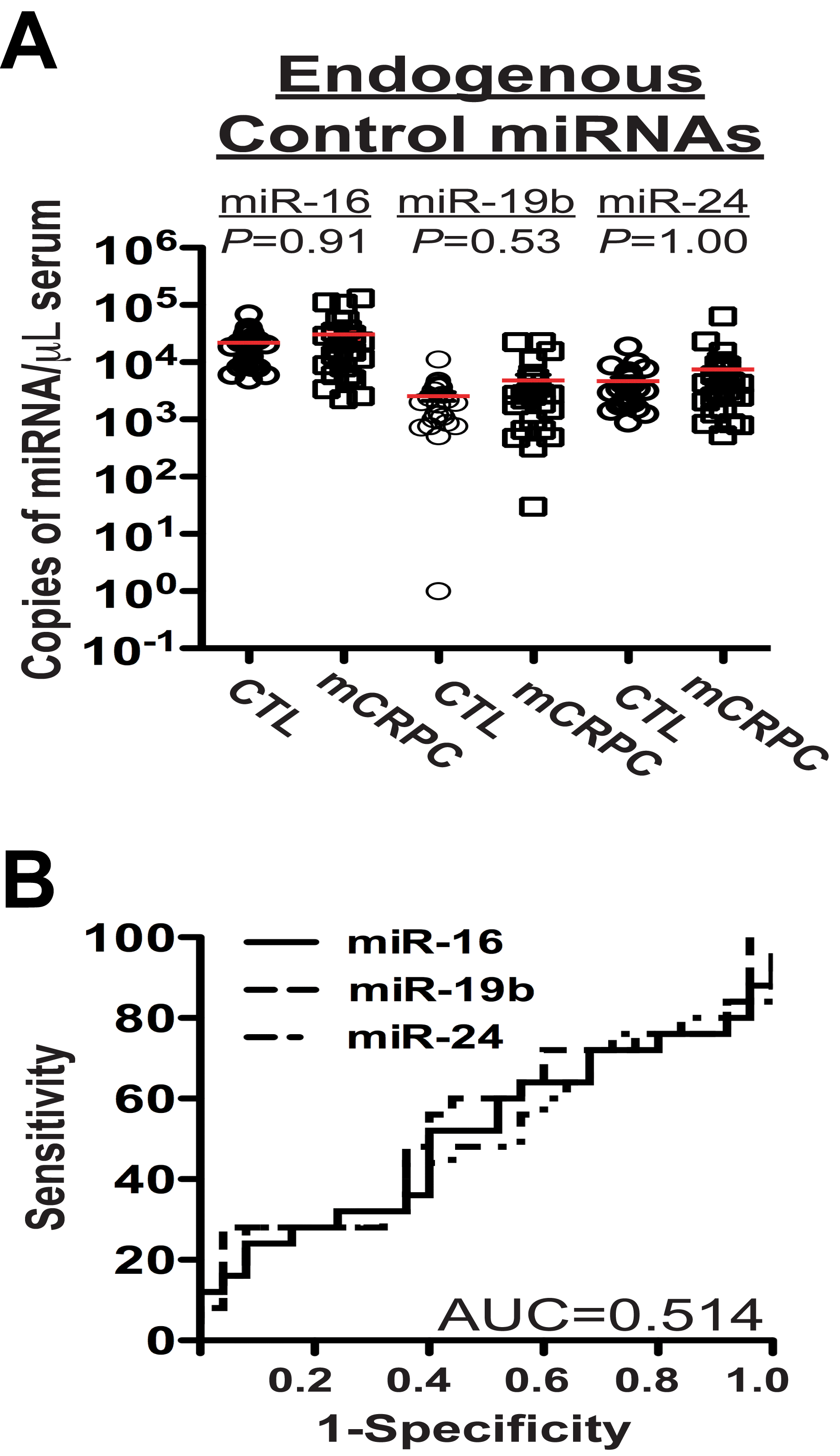

Supplement: Figure S1 — (A) miRNAs were measured in individual samples by TaqMan miRNA qRT-PCR (P value assigned by Wilcoxon signed-rank test), where miRNA abundance is given in terms of miRNA copies/µl serum. Red bars, mean +/− SEM of miRNA copies/µl serum for each group. (B) Receiver operating characteristic (ROC) curves plot sensitivity vs. (1 - specificity) to assess the ability of each serum miRNA to distinguish mCRPC and control sera. (TIF) [file pone.0069239.s001.tif]

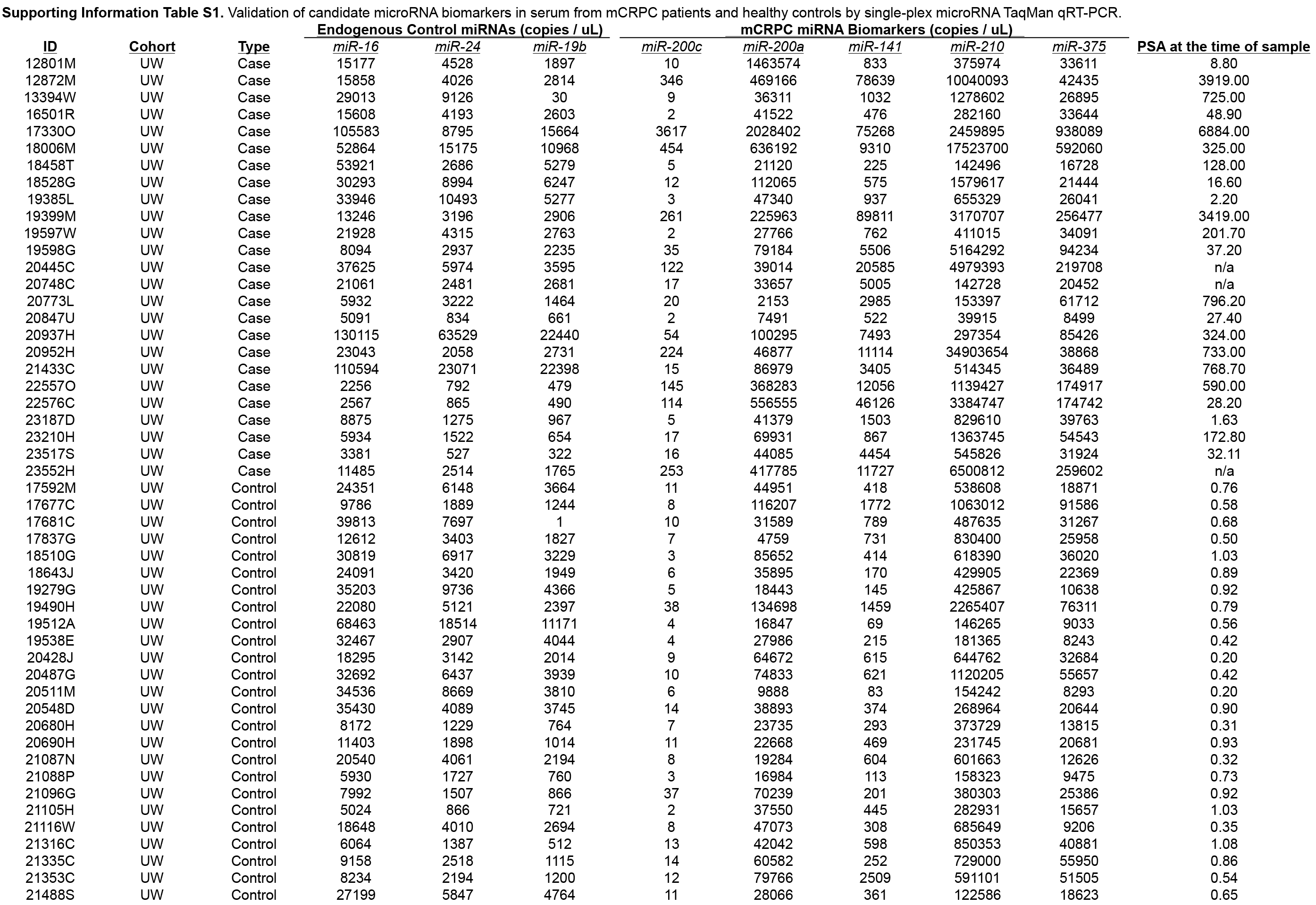

Supplement: Table S1 — (TIF) [file pone.0069239.s002.tif]

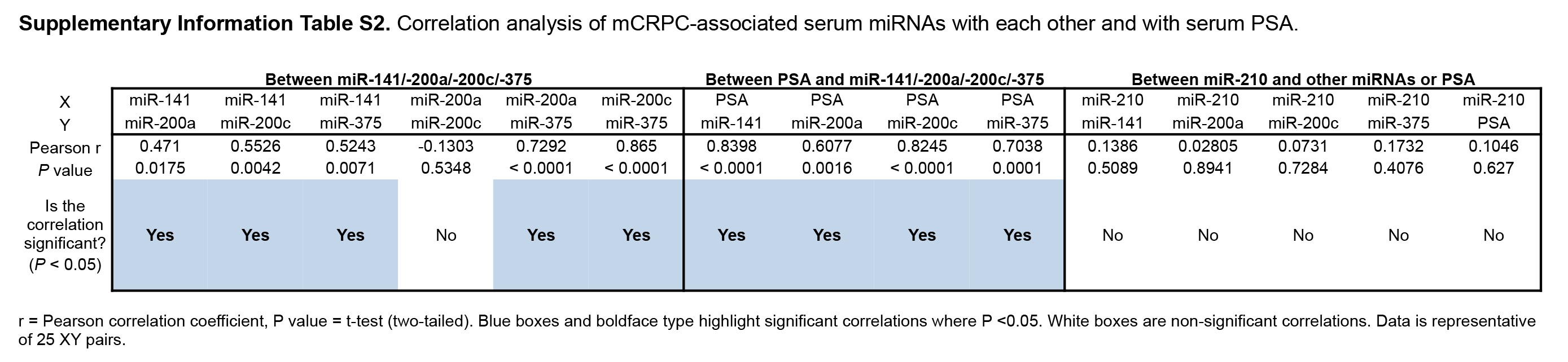

Supplement: Table S2 — (TIF) [file pone.0069239.s003.tif]

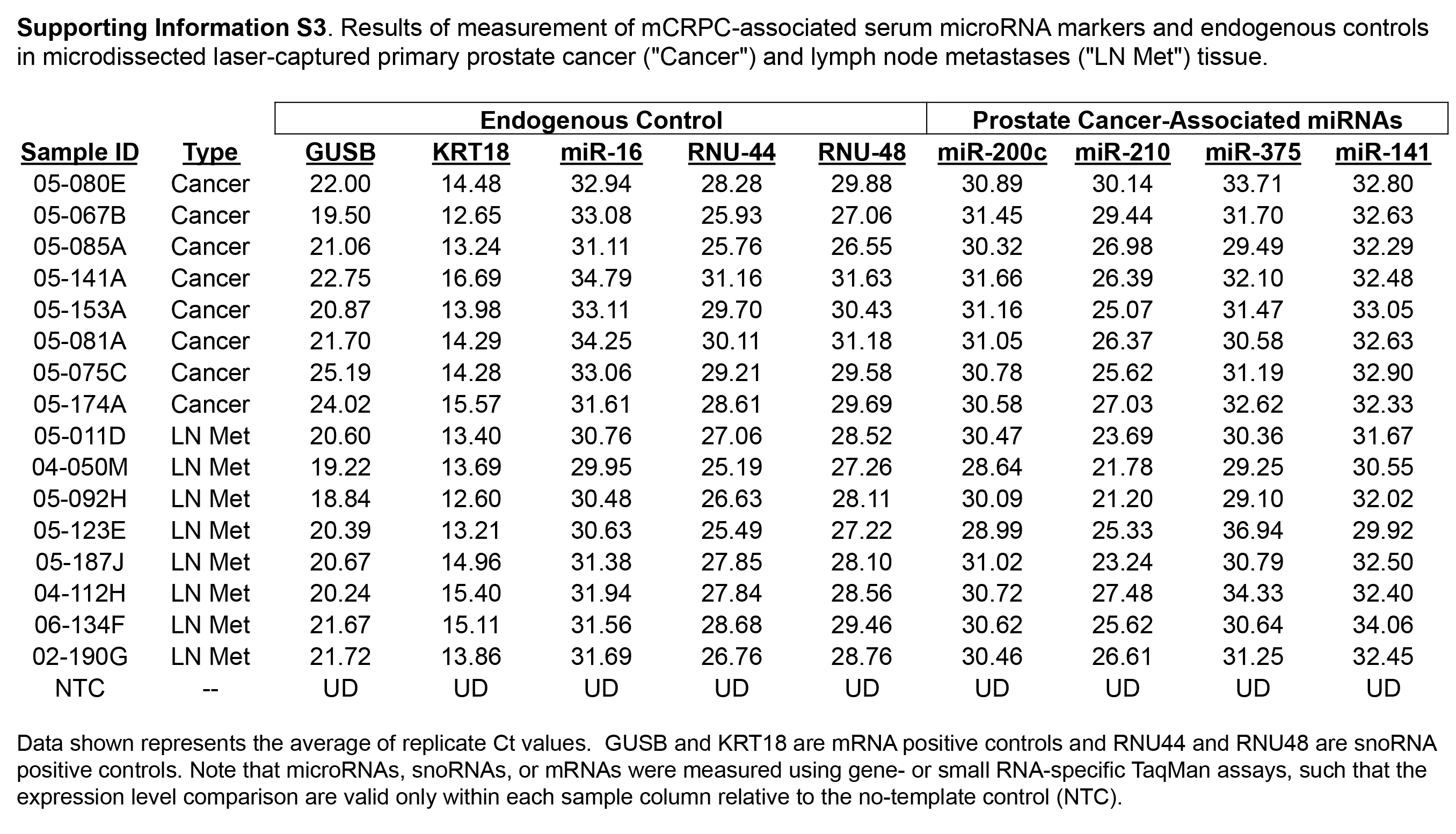

Supplement: Table S3 — (TIF) [file pone.0069239.s004.tif]
