# Supplementary material for: Circulating microRNA Profiling Identifies a Subset of Metastatic Prostate Cancer Patients with Evidence of Cancer-Associated Hypoxia
Source: PLoS One. 2013 Jul 30;8(7):e69239. doi: 10.1371/journal.pone.0069239 (PMC3728349; doi:10.1371/journal.pone.0069239)
Supplement: Table S4 — (PDF) [file pone.0069239.s005.pdf]

Supporting Table S4. Single-plex TaqMan assays used in this study

microRNA	ABI Product No.	Assay ID No.	microRNA TaqMan assay target sequence or gene expression amplicon
miR-16	4427975	000391	UAGCAGCACGUAAAUAUUGGCG
miR-19b	4427975	000396	UGUGCAAAUCCAUGCAAAACUGA
miR-24	4427975	000402	UGGCUCAGUUCAGCAGGAACAG
miR-100	4427975	000437	AACCCGUAGAUCCGAACUUGUG
miR-141	4427975	000463	UAACACUGUCUGGUAAAGAUGG
miR-148a	4427975	000470	UCAGUGCACUACAGAACUUUGU
miR-200a	4427975	000502	UAACACUGUCUGGUAACGAUGU
miR-200c	4427975	002300	UAAUACUGCCGGGUAAUGAUGGA
miR-210	4427975	000512	CUGUGCGUGUGACAGCGGCUGA
miR-222	4427975	000525	AGCUACAUCUGGCUACUGGGU
miR-375	4427975	000564	UUUGUUCGUUCGGCUCGCGUGA
miR-425-5p	4427975	001516	AAUGACACGAUCACUCCCGUUGA
GUSB	4331182	HS00939627_m1	not available
KRT18	4331182	HS02827483_g1	not available
RNU-24	4427975	001001	AUUUGCUAUCUGAGAGAUGGUGAUGACAUUUUAAACCACCAAGAUCGCUGAUGCA
RNU-48	4427975	001006	GAUGACCCCAGGUAACUCUGAGUGUGUCGCUGAUGCCAUCACCGCAGCGCUCUGACC

miRNA assays were designed against human miRNA sequences. Note that miRNA names and assay numbers provided are from the TaqMan qRT-PCR literature (Applied BioSystems), which uses miRNA nomenclature that predates the current miRBase release.
